# Case Report: Magnetic resonance imaging in rabies encephalitis

**DOI:** 10.4103/0971-3026.57214

**Published:** 2009-11

**Authors:** Arekapudi Subramanyaswara Rao, Dandu Ravi Varma, Mamidi Venkata Chalapathi Rao, Surat Mohandas

**Affiliations:** Department of Radiology and Neurology, Krishna Institute of Medical Sciences, 1-8-31/1, Minister Road, Secunderabad - 500 003, Andhra Pradesh, India

**Keywords:** Encephalitis, MRI, rabies

## Abstract

Rabies encephalitis is an invariably fatal disease characterized by typical clinical symptoms. Although the diagnosis of this condition can be made on the basis of the patient's history and the classical clinical presentation, neuroimaging may still play a role, especially for establishing an early diagnosis in cases with atypical presentations or when the history of animal bite is not forthcoming. We report the MRI findings in a case of furious rabies encephalitis and describe the utility of diffusion imaging in its diagnosis.

## Introduction

Rabies is one of the most feared diseases affecting human beings. It is invariably fatal once the clinical symptoms have set in. It is important to make an early diagnosis to enable the institution of public health measures. Literature regarding the neuroimaging features of rabies encephalitis is scanty due to two reasons: patients rapidly deteriorate following the onset of symptoms without a window in which to perform CT scan or MRI and the clinical presentation is often so characteristic that imaging may not be required. In this report we describe the MRI features in a case of rabies encephalitis and discuss the role of neuroimaging in this disease.

## Case Report

A 20-year-old woman was bitten on the hand by an unvaccinated puppy. Since the injury was a minor one, she ignored the incident and did not receive any prophylactic treatment. Nine days after the bite, she had prodromal symptoms in the form of fever, headache and malaise. Following this, she developed fluctuating consciousness, with irritability, episodes of agitation and frequent seizures. The episodes of agitation were characterized by hydrophobia and aerophobia.

On admission, she had rapid and shallow respiration. Cardiovascular examination was normal. On neurological examination, she had a Glasgow Coma Scale (GCS) of 12/15. The pupils were normal in size, symmetric and reacting briskly to light. The cranial nerves were normal. The muscle tone and deep tendon reflexes were normal and there were bilateral withdrawal plantar responses. Neck rigidity was absent. Because of her agitation, her higher mental functions could not be evaluated.

**Figure 1 (A-D):**
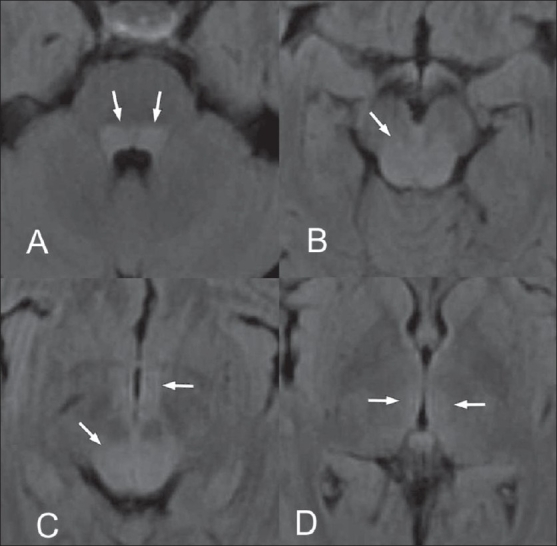
Axial FLAIR MRI images of the brainstem and thalamus reveal hyperintense lesions (arrows) involving the pontine tegmentum (A), most of the midbrain (B, C), hypothalamus (C) and medial parts of the thalami (D)

**Figure 2:**
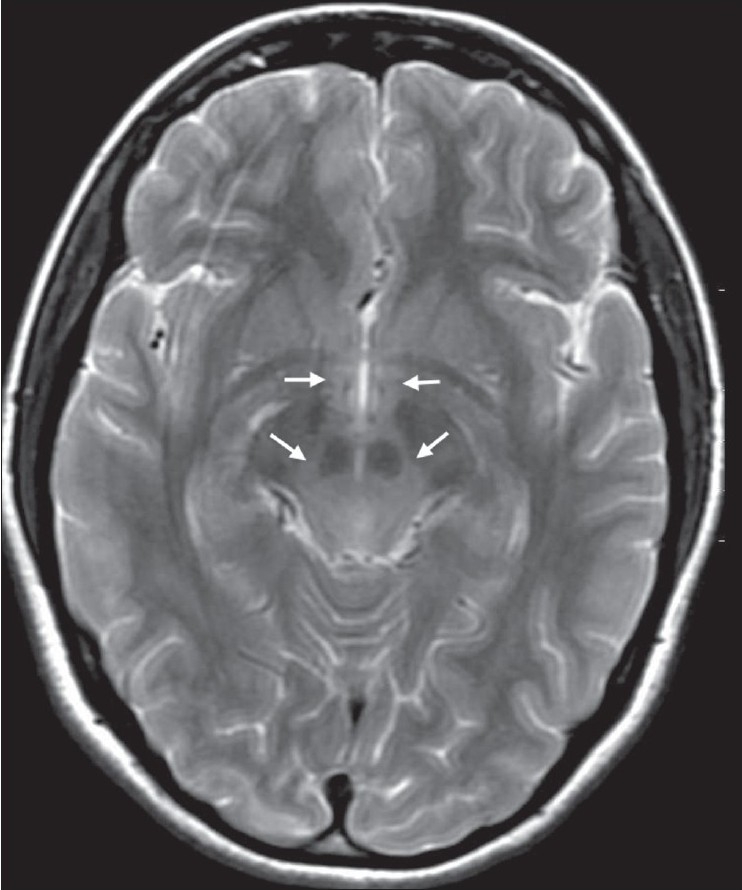
Axial T2W MRI image reveals hyperintensity in the midbrain and hypothalamus (arrows)

**Figure 3:**
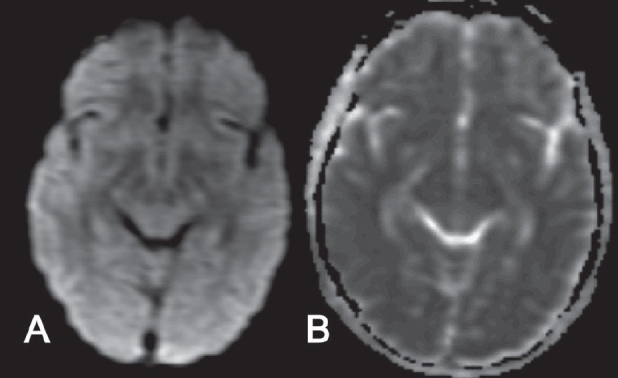
(A, B): Axial diffusion-weighted image (A) and apparent diffusion coefficient map (B) at the level of the midbrain do not show restriction of diffusion

Though the clinical presentation was classical and there was little doubt about the diagnosis, we decided to do an MRI of the brain to rule out other treatable causes of neurological dysfunction. On the sixth day after the onset of symptoms, an MRI of the brain, performed on a 1.5-Tesla scanner (GE Medical Systems, Wisconsin, MI, USA) under mild sedation revealed ill-defined hyperintense lesions involving the dorsal aspect of the medulla, pontine tegmentum, periaqueductal gray matter, collicular plate, as well as the central white matter of the midbrain. There were small foci of hyperintense signal in the medial aspects of the thalami and in the hypothalamus on both sides of midline [Figures 1 and 2]. The involved regions showed low signal on the T1W images. Diffusion imaging (b-value = 1000 s/mm^2^) revealed a mild increase in the apparent diffusion coefficients in the involved regions of the brainstem, hypothalamus and thalami [Figure 3]. There was a subtle mass effect with effacement of the aqueduct and third ventricle. The basal ganglia, cortical grey matter and white matter showed normal signal. The findings were considered consistent with those described in prior reports of rabies encephalitis.[1–3]

Blood glucose, serum electrolytes and renal and hepatic parameters were within normal limits.

In view of the public health implications of the disease, the patient was shifted to an institute for infectious and communicable diseases. Her neurological status continued to deteriorate, with depressed levels of consciousness and frequent seizures. On the eighth day of illness, her brainstem reflexes were found to be absent and supportive care was withdrawn after consultation with the family. Considering the characteristic clinical presentation and rapid progression of the illness to death, laboratory confirmation of the disease was not obtained.

## Discussion

Rabies is an acute, progressive encephalitis caused by a neurotropic RNA virus of the Rhabdoviridae family. Though it is extremely uncommon in the developed world, rabies is a significant public health problem in developing countries.[4] Of the 55 000 deaths due to rabies encephalitis that occur annually worldwide, 99% occur in Asia and Africa.[5] Though dog is the major vector and reservoir of the virus, several other mammals such as bats, wolves, jackals, raccoons, skunks and mongooses act as major hosts in different parts of the world. The virus is abundant in the saliva of the infected animal and is deposited in the wound inflicted on biting. The virus replicates in the muscle tissues at the site and subsequently infects the motor neurons and travels to the central nervous system by retrograde axoplasmic flow.[4,5]

After a variable incubation period and a prodromal phase that is characterized by burning, numbness, tingling and itching surrounding the site of bite, the patient presents with neurological symptoms. The acute neurological phase is characterized by two modes of presentation. The ‘encephalitic form’ is commoner and is characterized initially by hyperactivity, which soon progresses to periods of fluctuating consciousness. Phobic spasms, aerophobia and hydrophobia, triggered by puffs of air and sounds or even the mention of water are the hallmark of this form of the disease. In the ‘paralytic form’ of the disease, the typical cardinal signs appear late and are not prominent. A wide range of ‘nonclassic’ modes of presentation has also been described.[4,6]

Owing to the characteristic clinical presentation, the poor prognosis, and the difficulty in handling the agitated patient, imaging studies are rarely performed. CT scan findings have been reported to be non-contributory in most cases.[1,5,7–9] Awasthi *et al* in 2001 reported bilateral symmetric, non-enhancing hypodense lesions involving the basal ganglia.[10] Other regions of the brain involved by rabies encephalitis, such as the brainstem and hypothalamus are often poorly visualized on CT scans.

There have been very few reports describing the MRI features of rabies.[1,2,7,8,10,11] Laothamatas *et al* in 2003 described the MRI findings in three patients with the encephalitic form and in two patients with the paralytic form of the disease.[1] They reported ill-defined foci of mild hyperintensity in the brainstem, deep and cortical gray matter, deep and subcortical white matter and the hippocampi on T2W images. Both the paralytic and encephalitic forms of the disease have been reported to have similar distribution of signal changes on MRI.[4] Immunohistochemistry has shown that the distribution of these signal changes reflects the maximum concentrations of Negri bodies and rabies virus antigen.[12] Contrast-enhanced studies do not reveal enhancement of these structures in the early phase, while mild-to-moderate enhancement of the hypothalamus, brainstem and gray matter of the cord may be seen when the patient becomes comatose.[1] The brachial plexus is an exception and can show enhancement in the prodromal phase of the disease.

Definitive diagnosis of rabies requires laboratory confirmation of the presence of rabies antigen or rabies antibodies or the isolation of the virus from biological samples. However, rabies antigen and antibodies may not be detectable early in the course of the disease.[4] Also, the poor availability of these laboratory facilities may further delay the confirmation of rabies infection.

MRI may be used as an additional tool for the early diagnosis of rabies encephalitis. Demonstration of characteristic imaging features may allow early institution of public health measures: healthcare personnel can take special precautions while managing the patient and ensure optimal handling of body fluids and biological tissues.

MRI may also help differentiate this disease from other illnesses such as Japanese B encephalitis and other viral rhombencephalitides, acute disseminated encephalomyelitis, osmotic demyelination, hypoxicischemic encephalopathy, hypoglycemia and mitochondrial disorders—especially when the clinical presentation is atypical. The predilection of rabies for the brainstem, thalami and hippocampi; the reduced intensity of signal on T2W images; the absence of hemorrhages and the absence of enhancement during the acute phase of the disease may help in differentiating rabies from Japanese B encephalitis and other viral encephalitides. Rupprecht *et al*. have devised an algorithmic approach to the neurological manifestations and imaging features of rabies encephalitis and other common viral encephalitides.[6] The imaging features of rabies encephalitis on diffusion-weighted imaging have not been described. The involved regions of the brain in our case showed a mild increase in apparent diffusion coefficients. Thus diffusion imaging may also be used to differentiate this condition from acute encephalitis, ischemic encephalopathy and mitochondrial disorders—all of which would show restriction of diffusion.

Though the early diagnosis of rabies does not have any impact on patient prognosis, it does enable prompt institution of public health measures. Limiting contact with the patient and identifying the animal responsible for transmission may prevent the spread of infection to other individuals.
